# “To Work Just Like Anyone Else”—A Narrative from a Man Aging with Spinal Cord Injury

**DOI:** 10.3390/healthcare5040087

**Published:** 2017-11-09

**Authors:** Ulrica Lundström, Margareta Lilja, Gunilla Isaksson

**Affiliations:** 1 Department of Health Science, Luleå University of Technology, S-971 87 Luleå, Sweden; margareta.lilja@ltu.se (M.L.); gunilla.isaksson@ltu.se (G.I.); 2 Department of Health Sciences, Lunds University, Lund S-221 00, Sweden

**Keywords:** aging, meaning, occupation, secondary health complications, spinal cord injury

## Abstract

People aging with spinal cord injury (SCI) develop medical problems commonly associated with the aging process at a younger age than the general population. However, research about how the life story changes and how meaning will be experienced in occupations is lacking. The aim was to describe and offer an explanation of how a man experienced meaning in everyday occupations while aging with an SCI. Four narrative interviews were performed over a four-year period, with a man in his fifties, who lived with SCI for 39 years. The narrative analysis generated an overall plot, named “To Work Just Like Anyone Else,” and gives a picture of his experiences, thoughts, and reflections about meaning in occupations, from when he became injured to the present, and in relation to his future. His life story is characterized by secondary health complications, and his experiences of negotiating with the aging body and making choices to continue working. Further, how occupational risk factors, e.g., imbalance, alienation, and deprivation, occur as a result of lack of rehabilitation and support from social systems is addressed. Future research should explore how rehabilitation and social systems can support people aging with SCI to experience meaning in everyday occupations and to have balance in everyday life.

## 1. Introduction

Aging is an inescapable phenomenon for everyone, and yet in the general population, people usually remain independent in their advanced years [1]. The image of modern senior citizens shows an active and self-realized lifestyle, being engaged in meaningful activities, with the most distinctive feature being that they are independent and autonomous, without any significant illnesses or disabilities [2,3,4]. This period has been called the third age, a term introduced by Laslett [5], referring to a period in life between the second age of professional work and family responsibilities, and the fourth age. The latter is characterized by dependency, disease, and disability, and occurs usually when the general population in the Western world is in their 80s [1,5,6], although that is in contrast to the aging of people with disabilities, such as spinal cord injuries (SCIs). 

People aging with SCI develop medical problems commonly associated with the aging process at a younger age than does the general population [7,8,9]. In addition, a wide range of secondary health complications have been noted, and a majority of these occur with higher frequency among those with longer SCI duration [10,11]. Research describes that, as a result of physical deterioration, people with SCI may need increased help as early as their early fifties or sixties, depending on the severity of the SCI, in the areas of personal care, such as transfers, bathing/showering, and dressing [9,12]. Even though the major rehabilitation goals of people aging with SCI are maintenance of health, maximal functioning, and quality of life [13], this topic has been sparsely studied [7,14], in contrast to rehabilitation for individuals who are newly injured [15,16,17,18,19,20,21].

To deepen our understanding of and address this knowledge gap, through several studies [22,23,24,25] we have generated knowledge that can be valuable for improving rehabilitation interventions and that can guide work through policy recommendations for social service systems, in order to enable participation in occupations for people aging with SCI. Occupations which can be seen as all the things that people do and engage in, alone or together with others in their everyday lives [4].

The previous studies have focused on how participation in occupations changes over time, as a result of secondary health complications and the increased need to save strength and energy [22,23,24]. In addition, it has become clear that support systems (including rehabilitation, personal assistance, and assistive devices) do not always support participation in occupations [23,24,25]. All of these factors together, caused anxiety, uncertainty, and frustration, as everyday life has turned out to be a complex daily struggle. However, the previous studies raised questions regarding how a person’s life story changes and how meaning will be experienced in occupations while aging with an SCI [22,23,24,25]. 

No matter where in life a person with SCI is situated, rehabilitation should support the individual’s ability to participate in daily occupations, find meaning in daily life, connect to places and people, and belong to and be trusted by others [26]. As a rehabilitation profession, occupational therapists have an interest for how occupational engagement constructs meaning and can thereby contribute to a life worth living and a sense of health [27,28]. For this study, we chose a definition of meaning that focuses on how people experience meaning in their occupations [27,29,30,31], and in short, meaning has been described as having four dimensions: *doing*, *being*, *becoming* [30], and *belonging* [31]. 

In order to explore experiences and changes of meaning in occupations, along with enhancing the understanding of how aging with an SCI can change a person’s life story [32], we argue for the value of using a narrative method as has been described by Mattingly [33]. A narrative method has proven successful in earlier research among people with SCI [34,35,36], and there are a number of advantages to using this method. For example, listening to the stories of individuals with disabilities can reveal the experiences of occupations in the individual’s life and what his/her expectations are for the future [32,37,38]. Thus, narratives may challenge our assumptions and enable us to deepen our overall understanding of aging with SCI. Based on these arguments, we have used one man’s narrative to examine his experiences, thoughts, and reflections about meaning in occupations while aging with an SCI, in order to obtain a profound understanding of his life story. The aim of the study was to describe and offer an explanation of how a man experienced meaning in everyday occupations while aging with an SCI. 

## 2. Materials and Methods 

### 2.1. Design 

This study is part of a larger research project with the aim of exploring participation in everyday life while aging with a traumatic SCI [25]. To help achieve this aim, we have chosen to undertake a narrative method. Narrative interviews [39] were used to capture a man’s life story about his meaning in occupations while aging with an SCI and to reach both a retrospective and prospective perspective. To analyze the data and offer an explanation of how he experienced meaning in occupations, in relation to events, a “narrative analysis of eventful data” as described by Polkinghorne [40] was used. The use of a narrative method enabled us to focus on the ongoing processes in his everyday context. The study was approved by the Regional Ethical Review Board in Umeå, Sweden (2012-236-31M).

### 2.2. The Participant 

William (a pseudonym) was selected through purposeful sampling [41] from the eight persons included in our earlier study [23]. These participants were recruited based on the following inclusion criteria: (1) traumatic SCI, (2) tetraplegia, (3) over 40 years of age, (4) a minimum of 10 years post-injury, (5) a place of residence based on geographical location to enable personal encounters for the interviews, and (6) participation in a so-called “retro course”, arranged by the national non-profit organization RG Active Rehabilitation in Sweden (this organization has a special interest in active rehabilitation and provides opportunities to people with SCI, both to meet role models who have experienced living with a comparable type of injury and to participate in organized camps with different themes). The course was designed as a client-centered and goal-oriented program in order to provide information about secondary health complications and how these can be prevented in order to maintain and/or increase quality of life and independence while aging with an SCI. The criterion for selecting William was the richness of his narrative in the first interview, since he experienced an increasing imbalance in his everyday life and therefore faced a changing life story. The first author contacted him by email and explained the aim of this study. He agreed to participate, and thereafter a phone call was made to give oral information and to answer any questions before making the appointment for the interviews. 

### 2.3. Data Collection

The first author interviewed William four times (two face-to-face and two phone interviews) over a four-year period. The face-to-face interviews lasted between 2 and 2.5 h each, and took place in his home (based on his decision); the phone interviews lasted about 1.5 h each. Narrative interviews were conducted according to the description of Reissman [40]. Throughout all four interviews, the narratives were guided by open questions, which allowed William to give free responses; this technique captured his story from when he got injured until the present, together with his thoughts for the future. This process of data generation facilitated natural conversations and interactions, and created opportunities for William to reflect on and link his current situation with previous experiences and future expectations. It also provided the first author with opportunities to ask questions to deepen understanding and achieve rich data. All interviews were recorded digitally with an MP3 player and were transcribed verbatim.

### 2.4. Data Analysis

The analysis was conducted according to the “narrative analysis of eventful data”; the outcome of such a method is a life story or a storied episode of a person’s life [40]. The first step was to obtain a sense of the overall data; all the transcribed interviews were read several times. During the second step, the first author started to search for significant events in the man’s story that matched the purpose of the study. The significant events were then separated from the text and arranged chronologically, in order to create a story with a beginning, a middle, and an end. In the third step, a process called “narrative smoothing” [42] was used in order to exclude irrelevant data that did not contribute to the story. The events were used to outline a story that could give a possible explanation to the man’s experiences of meaning in occupations while aging with an SCI. In the fourth step, the first and third authors compared the outlined story to the unused parts in the narratives, i.e., events not used, to determine whether any of them could further enhance the understanding of the changes that occurred in the man’s life. In the fifth and final step, the first author conducted a to-and-fro movement between the emerging story and the unused parts, with the aim of gaining a deeper understanding of the complexities and finding a suitable plot. Throughout the two last steps, some events were excluded and others were added to be able to tell his story. Our final story will be presented as an overall plot, named “To Work Just Like Anyone Else,” and is divided into five parts, based upon significant events in his life story. 

## 3. Results 

William’s life story gives a picture of his experiences, thoughts, and reflections about meaning in occupations, from when he got injured to the present, together with thoughts in relation to his future. To tell his story, we have used an overall plot—“To Work Just Like Anyone Else”—to illustrate the importance for William of being a worker. The story is characterized by the onset of, and increasing physical deterioration due to, secondary health complications, and Williams experiences of how he is negotiating with his aging body and making choices in order to continue to be a worker. 

### 3.1. A Work on the Same Conditions as Others

When William was 16 years old, at the end of the seventies, he described how he dove into shallow waters and sustained a complete (“complete” refers to severity of the injury, meaning having no motor or sensory function below the injury level) SCI at the C4–5 level (”C” refers to cervical vertebrae, and “4–5” states the neurological level of damage). At the end of the first two years of initial rehabilitation, William realized that he would not improve his physical functions to such a degree that it would be possible for him to participate in occupations without help. This insight led him to decide to quit rehabilitation and go back to school, since he really enjoyed studying and found it meaningful. This was the first step in taking control and finding a new structure for his everyday life. He educated himself in the field of computers and information technology, a choice based on his belief that, by doing so, he could get employment just like anyone else. As he said, “the computer has been a tool in my work, and not a technical aid.” This education did make it possible for him to compete with others on the same conditions when applying for employment; after approximately five years of studying, he got his first job as a software engineer after replying to a job advertisement. 

William needed a lot of assistance in several occupations throughout everyday life. A couple of years after the injury, he realized that he had no influence over the assistance that he was provided by the social system, and he expressed his thoughts on the matter:

“I met a new person in the shower every day! The monkeys in the zoo can choose which animal keeper will feed them, but if you’re in need of help, you just have to accept the help and the people that are coming to you.”

This experience resulted in a desire to make a change, William and a few other people with disabilities started a company to administer their personal assistance. Being in control of his personal assistance, provided him with energy and strength to engage in occupations that were meaningful for him.

### 3.2. Life Itself and Work Are Running Smoothly

William experienced his everyday life as running smoothly and in some ways static for a period of about twenty years. After his first employment following the SCI, William worked part time (50%) as a software engineer, and he found his work to be meaningful. At that time of his life, he believed that he had the capacity to work more, but the rigidity of the rules made it impossible to do so. Throughout the years, he changed companies twice; as a result, the job assignments shifted to some extent, but they have all been a part of his professional development. 

Since the beginning of 1990s, William and his wife have lived in a house; early on, he describes how they actively made decisions together regarding the support they needed in order to minimize the involvement of personal assistants in their everyday life. This was reflected in William´s experience:

“It was an obvious choice for us and it became even more obvious when our son was born… it felt so awkward to have someone sit here and wait a couple of hours just to help transfer me to bed… Instead, my wife helps me with that and it only takes approximately 10 min… so this way of actively choosing makes our everyday lives work smoothly.”

William and his wife had allocated household chores between them; he, with help from personal assistants, was responsible for cleaning and doing the laundry in the home. Later on, he also helped their son with his homework and gave him rides to a variety of occupations, such as soccer practice or visiting friends. He experienced this last chore to be an obvious responsibility, since he was able to be independent in all the different phases of driving his adapted van. This had not always been a reality; William remembers being frustrated when driving an adapted car with an ordinary driver’s seat early on after the injury. Thus, he was able to travel longer distances in the community, but still needed help with transfers between the manual wheelchair and the car. For that reason, it was an unforgettable moment when, in the beginning of 1990, William was introduced to the option of using an electric-powered wheelchair as a driver’s seat in an adapted van. A whole new world opened up for him, as he was able to drive his van and participate in necessary and desired occupations for him, in the community yet on his own terms. 

William enjoyed spending time with family and friends, traveling, dining out, attending cinemas/theater, reading, watching TV, and doing computer activities. He described how everything in his everyday life seemed possible and he never hesitated to participate in different occupations. For example, when he travelled to foreign countries together with his family, he had a strategy which he explained:

”It was just to do it like the last time and everything would work out just fine… or if something would happen, for example to the wheelchair, there was always a bike store or something that could help me out.”

Altogether, William expressed that, during this period of his life, he reached a comfort zone where he knew how to compensate for the SCI and was able to engage in desired occupations. He experienced hope for the future and could see opportunities to continue to develop, both professionally as a worker and personally as a parent and a spouse. 

### 3.3. A Sudden Need to Start Reorganizing Work 

William described how, after approximately twenty-five years, he noticed that his daily routines started to take extra time, and how small pressure sores appeared where he earlier just experienced redness. In addition to this, he started to feel queasy, had nausea, and experienced fatigue during every day. All these changes to his health caused difficulties in focusing on the job assignments. It was nearly a year before William was diagnosed with sleep breathing disorder/apnea, and by this time he had decided to reduce his working time from five to four days in order to have an extra day off for recovery. He experienced a relief to know what caused his problems, although he still expressed frustration:

“I now have a ventilator at night… I´m not totally dependent on it, but if I don´t use it, it has its consequences, and I do get problems working or participating in any other occupations the day after. So I guess it´s a hands-on example that my physical functions are starting to deteriorate and that I now need one more thing/assistive device/technical aid to be able to function as optimally as possible in everyday life.”

William describes how, after another two years, he encountered new problems when performing his job assignments, even though he was working part-time divided over four days per week. He experienced himself as ‘butter-fingered’ and no longer capable of writing texts like he used to, and he made more errors while typing on the computer. Even though his handwriting had only been readable to him, it now became increasingly difficult for him to read it himself. Once again, he experienced an increasing fatigue and a sense that the workdays started to be more demanding, with not enough time to recover from work. In order to continue working, William realized he needed to reduce his working hours further via the sick-leave system and divide his working time into three days a week, with a day of rest between each. By doing so, he sincerely hoped that he would be able to continue working but he started to worry. William expressed his concerns for the future:

”It´s obvious that the path of decline has started and I don´t know how steep the path will be… or how fast it will be… or which symptoms will be next in line for me to experience… Still, it´s obvious that I have entered in to this path of decline and I start to see that there will be problems for me to be in control of my everyday life.”

William experienced how the onset of secondary health complications threatened his sense of himself as a worker, which in turn raised worries about the future and not being able to continue to work. Even though he also mentioned how the increasing health problems made it difficult to participate in other meaningful occupations, it was his concerns about work that troubled him the most. In the midst of all this, he felt alone with his concerns, with no obvious professional to turn to within healthcare or rehabilitation to get advice and support. 

### 3.4. Trying to Combine Work with Life Itself

After four years with three workdays per week and more or less difficulties managing it, William explained how everyday life had become a balancing act, with decreased energy for work, but also for family and friends. He described an increased need to rest in order to be, as he says, “fit to fight for work.” William continued:

“I don´t know what to do differently… even when I think of the days when I come home from work more or less exhausted… what would I have done differently? I have no answers and that bothers me because I think of myself as solution-driven… now I begin to glimpse a problem: how will I manage to work in a few more years at this pace, while continuing to be the person William and not the patient William?”

William realized the need to turn down engagement in occupations. A change that started with just a few occasions had now become a natural part in his everyday life in order to save energy for work. He could no longer be engaged in occupations like spending time with friends, or going to a restaurant, theater, or cinema together with his wife and/or son during the week nights when he had been working. When prioritizing which occupations to engage in, he tries not to give up occupations that are important to the family, and he has adopted a strategy that “less is better than nothing.” William describes about how he sincerely hopes that this wisdom is going to work in the long run:

”Of course, it´s sad that I´m not able to help my son to the extent that he might need… Instead of helping for two hours, I can help him for half an hour… or saying no to an activity that my wife has planned for a week night… or when an evening ends up shorter than we both had hoped since I´m feeling exhausted…”

Even though William knew that he had to prioritize among which occupations to participate in, both for himself and his family, he could not really accept this reality. He explained that, by not thinking about it, it seemed somewhat easier to do. On the other hand, every now and then, he reflects that it might have been easier for him than for others to actively choose not to engage in occupations. William explained:

“Sometimes, I enjoy being by myself… home alone in a physical environment where I can be rather self-driven… I guess it would have been more difficult if I did not find activities like reading, watching TV, or spending time with the computer as meaningful… so now I can see them as an alternative to activities that can be too demanding for me.”

William gave an example of how small events in his everyday life reflected his physical deterioration: when he wore a thick knitted sweater on a cold winter day, he suddenly experienced difficulties driving and parking his van, since it further restricted his arms’ limited strength and range of motion. This experience stirred up strong emotions, since driving was associated with a tremendous amount of freedom. He expressed his concern about what would happen if he were unable to drive his adapted van in the future, since that would seriously interfere with his ability to get to work or participate in desired occupations in the community. 

William’s thoughts around the choices he made throughout his life give a picture of a solution-driven person. As problems with engaging in occupations occurred more frequently, he experienced the need to be more creative to solve problems, since he no longer had the same physical resources as earlier. William described:

“I can no longer trust my body and my capacity. It´s not the same from year to year… so now I can’t rely on doing the activity in the same way as the last time and know that it’s [going to] work… Now I have to plan several steps ahead and be prepared for a variety of things that can happen… something I never would have thought of earlier.”

At this point in life, William experienced frustration and ambiguous feelings; because he was no longer in control since his physical capacity was constantly changing, he felt like he was no longer in charge of his everyday life. 

### 3.5. When Work Will No Longer Be an Obvious Part of Life

Recently, William experienced several hospital stays, and he expressed his concern: “What´s going to happen next… How decrepit can one be and still have a well-functioning everyday life with work and a social life?” 

He struggles to find the right level of work that will enable him to experience balance once again in his everyday life. He is trying to use his time wisely at work, and he takes the opportunity to rest in his electric-powered wheelchair. He informed his closest manager and colleagues that his physical capacity is changing, and they understand his situation. At the same time, it is difficult both for them and for William to know what to do about his situation to enable him to continue to be a worker. William wondered whether it would be a good idea to reduce his working time further, to 25% of a customary workweek, but this raised questions: what would he be able to accomplish in such a short time, and would the employer accept that work time at all? Once again, he is becoming aware of the rigidity that the social system displays when there is no possibility of an individualized work time based upon one’s ability. William knows that he can retire early from work, but this is not an option today: he explained:

“It sounds so boring to feed the doves in the park… It’s like being in between a rock and hard place. Should I be bored as a retired person or be worn out as a working tetraplegic person? If I let my body get the space it seems to need in my everyday life, my mind would be bored… It’s a balancing act on a high level, and I guess a time will come when I fall off the line and the problem is solved…”

The question William is asking himself is: “What to do when work is no longer an obvious part of everyday life?” He expressed the need to find something meaningful to engage in:

“I’m not the kind a person that can kill days, weeks, and months by just spending time in front of the TV or the computer… I would like to do something that can contribute to others… and also to find a balance… where I can manage to experience meaningful occupations and still handle my aging tetra body and its physical limitations…”

William wonders whether it is possible to find these experiences in any other occupations besides work. For him, work means a place to go to and not being isolated at home, a place where his competency is needed and where he can be creative, collaborating with colleagues to solve problems. He thinks it would be easier to make the decision to retire if he already had found a meaningful occupation to engage in. Today, William has no idea what to do, and he is realizing how energy-consuming his balancing act of everyday life is, which probably makes it even harder for him to find an alternative occupation to work. William’s narrative ends with a citation that gives a picture of the frustration, along with the lack of choice and control, he is experiencing now in his everyday life:

“I want to have energy for work, for myself and for my family, so there are many threads that draw together… and in the middle of this is the aging tetra body that ultimately decides… that doesn’t seem to be the least negotiable… and it is disturbing… I got used to the body not cooperating, but now it has started to strive against me…”

## 4. Discussion

The overall aim of our research project is to explore participation in everyday life while aging with a traumatic SCI. This study adds to that aim, as it sheds light on different perspectives of meaning in everyday occupations while aging with an SCI. William’s life story shows how he negotiates with his aging body and makes choices in order to continue as a worker and to find meaning. The story is also characterized by physical deterioration due to secondary health complications, implicating how occupational risk factors such as imbalance, alienation, and deprivation occur as a result of lack of rehabilitation and support from social systems, which in turn can further threaten his health. 

In contrast to the balanced everyday life William experienced for almost two decades, he is now experiencing an increasing imbalance. Imbalance is one of the occupational risk factors together with alienation and deprivation, all of which have been identified in relation to the dimensions of meaning [27,29], and are associated with the occupational justice movement [43]. Occupational alienation can be described as feelings of isolation, emptiness, and a lack of sense of being, whereas occupational deprivation is the lack of not only choice and control but also diversity of occupations [44]. These risk factors can occur in all kinds of occupations, but they occur perhaps especially in work, since it makes up a significant part of life for many individuals, particularly in Western society [45]. William’s desire to have a career on the same conditions as persons without disabilities is also in line with earlier research, which shows how work is viewed as a meaningful occupation, since it brings structure to an individual’s life and can enhance feelings of an identity and self-worth through the sense of being valuable and competent. It also adds to the importance of belonging to a social network, such as with colleagues at a workplace [27,29,46]. To not be a part of work can therefore have a great impact on meaning in everyday life for persons aging with a disability such as SCI. At this point, the structure of the sickness compensation system in Sweden, with its fixed steps in relation to working capacity (0%, 25%, 50%, 75%, or 100%) and its rigidity, can be a barrier for supporting people to continue to work. The system thus increases the risk for William or others in a similar situation, to experience occupational alienation and deprivation. When experiencing these occupational risk factors, stress can develop and further threaten a person’s health [3]. Based on these factors, we argue for the possibility to have an individualized work time based on one’s ability. 

The secondary health complications that William is experiencing are not unique to him; they are in line with earlier research [8,24,47,48,49] and can be addressed as a sign of accelerated and/or premature aging [7,14]. The onset of secondary health complications and deterioration of his physical capacity affected his engagement in meaningful occupations and threatened his sense of himself as a worker. Thus, it changed his feelings of himself as an occupational *being*, causing frustration and ambiguous feelings due to a lack of choice, control, and meaning in everyday life; this in turn raised worries about the future. We interpret these experiences as a result of his fear of not having the opportunity to continue to develop as a worker, which is in line with earlier findings of decreased possibilities of *becoming* [3,50,51]. The meaning that William experienced in work can be related to *doing*, *belonging*, and *becoming*, where belonging has been described as especially important for a person’s being [52]. The relationship between these dimensions has only been sparsely explored in empirical studies [51], although here we can see a relationship between *being* and *belonging*, as work gives him feelings of connectedness and a wish to continue to develop both as a person as well as a team together with his colleagues. These feelings are not specific to William or other persons aging with disabilities; it has also been found in an earlier study of people reaching retirement age [53]. The difference in our findings could be the lack of choice and control combined with the uncertain future that William is experiencing as a result of physical deterioration. 

In terms of for example rehabilitation and surveillance recommendations for people aging with an SCI, a gap of knowledge has been described by Groah and her colleagues [7,14]. This is in line with our findings, since William is once again in need of rehabilitative interventions, but this time he feels alone, with no obvious profession to turn to within healthcare or rehabilitation. This can be seen as a barrier for aging successfully with SCI. Based on the results of this study and our previous studies [22,23,24], we suggest a lifelong rehabilitation program with regular follow-ups by a multi-professional team for people aging with an SCI [54]. The frequency of the follow-ups should increase with time, and efforts should be made to screen their everyday lives to create/re-establish participation and a balance among their meaningful occupations. This is in line with previous research that describes how replacing work with other meaningful occupations is critical to experience a good life as a retiree [55], something that can be viewed as more important for people aging with SCI. In addition, our findings support earlier research that shows how interventions need to focus on whichever dimension of meaning might be in the foreground [29,30,56], and not only on *doing* which has been the case in rehabilitation for many years [29]. 

We also want to highlight the rather long period of time in which William experienced his life as static and running smoothly, something that is in line with a previous theory about aging with a disability [57]. In order for this to be a reality, it is crucial that support systems such as personal assistance, assistive devices, and an adapted car/van are working appropriately. During this time, William was able to engage in meaningful occupations for him, such as work, traveling, and spending time with family and friends. Thus, it was a balanced interaction between *doing* and *being* that led to a realization of who he was as well as having hopes and ambitions for the future, something that we interpret as *becoming* [3,29,51]. *Becoming* has been described as a process with cycles of achieving goals and then setting new ones [58]; it is an ongoing process throughout a person’s lifetime and is important for experiencing meaning in everyday life and for personal growth/development. 

William’s story can be viewed as a small piece in the puzzle of aging with an SCI, and it reveals a need for further research on how rehabilitation and social system can support people aging with an SCI to experience meaning in everyday occupations and to have balance in everyday life.

## 5. Methodological Considerations

To enhance trustworthiness and improve credibility [59] for this study, we provide an expanded description of the method and of the process of data collection and analysis. The first and last authors had a constant dialogue during the analysis process, and the second author asked analytical questions to ensure that the first and last authors adopted a self-critical stance. Representative quotations from the transcribed text were used to show how the identified plot covers the data. The results in this study should be viewed in light of the limitation that narrative data is situated within the communicative context in which it is gathered. At the same time, it is important to remember that narrative data should be seen as a tool in which alternative understandings can be explored and deepened. Furthermore, the results from a narrative study can serve as a foundation for further research. 

## 6. Conclusions

The findings shed light on different perspectives of meaning in everyday occupations while aging with SCI. It further explains how physical deterioration, due to secondary health complications, is creating an everyday life that is characterized by negotiation with the aging body and making choices in order to continue as a worker and to find meaning. Occupational risk factors such as imbalance, alienation, and deprivation occur as a result of the lack of rehabilitation and support from social systems, which in turn can further threaten one’s health. The findings implicate the need for lifelong rehabilitation, and we propose regular follow-ups, by a multi-professional team, that increase over time, together with well-functioning support systems from society. All professionals should have a client-centered approach and the overall aim should be creating/re-establishing participation and a balance among meaningful occupations for people aging with SCI. 
